# Stay or go? Exploring physician turnover in European Hospitals–Evidence from the METEOR survey

**DOI:** 10.1371/journal.pone.0337287

**Published:** 2025-11-21

**Authors:** Laura Maniscalco, Marco Enea, Peter de Winter, Neeltje de Vries, Anke Boone, Olivia Lavreysen, Kamil Baranski, Walter Mazzucco, Adriano Filadelfio Cracò, Malgorzata Kowalska, Szymon Szemik, Lode Godderis, Domenica Matranga

**Affiliations:** 1 Department of Health Promotion, Mother and Child Care, Internal Medicine and Medical Specialties, University of Palermo, Palermo, Italy; 2 Child and Youth Institute, KU Leuven, Louvain, Belgium; 3 Department of Development and Regeneration, KU Leuven, Louvain, Belgium; 4 Department of Pediatrics, Spaarne Gasthuis, Haarlem and Hoofddorp, The Netherlands; 5 Department of Internal Medicine, Spaarne Gasthuis, Hoofddorp, The Netherlands; 6 Spaarne Gasthuis Academy, Hoofddorp, The Netherlands; 7 Department of Public Health and Primary Care, Centre for Environment and Health, KU Leuven (University of Leuven), Leuven, Belgium; 8 Department of Epidemiology, School of Medicine in Katowice, Medical University of Silesia, Katowice, Poland; 9 Department of Planning, Programming, Strategic Control and Management Control, Local health Authority of Agrigento, Agrigento, Italy; 10 IDEWE, External Service for Prevention and Protection at Work, Heverlee, Belgium; University of Porto Faculty of Medicine: Universidade do Porto Faculdade de Medicina, PORTUGAL

## Abstract

According to the World Health Organization (WHO), in 2022 there was a shortfall of approximately 1.2 million doctors, impacting healthcare system and patient care. Understanding turnover intentions is crucial for managing the healthcare workforce and ensuring continuous, and high-quality patient care. This study investigates the prevalence of physicians planning to leave their hospital or the profession, and risk factors such as job demand, resources, satisfaction, and burnout across four European countries. A cross-sectional multicenter study was conducted in eight hospitals across Belgium, the Netherlands, Poland and Italy, including both academic and non-academic institutions. Data from Poland were excluded due to a low response rate, to preserve respondent anonymity. Multivariable logistic regression analyses were performed, adjusted for country, demographics, and work context, using significant variables from the univariable analysis. The overall intention to leave the hospital was 16.5%, with the highest rates in Belgium (19.6%) and Italy (19%), and the lowest in the Netherlands (9.8%). The intention to leave the profession was 9.1%, with the highest rate in the Netherlands (16.1%), followed by Belgium (6.3%) and Italy (5.7%). Physicians at higher risk of leaving the hospital were younger (adjOR = 0.90, 95%CI = 0.86–0.93), lacked colleague support (adjOR = 3.18, 95%CI = 1.06–9.36), and were dissatisfied with job prospects (adjOR = 2.38, 95%CI = 1.02–5.54) and overall work (adjOR = 2.71, 95%CI = 1.09–6.69). Those more likely to leave the profession were from the Netherlands (adjOR = 4.14, 95%CI = 1.62–11.4), surgeons (adjOR = 2.90, 95%CI = 1.22–6.78), working in non-academic hospitals (adjOR = 2.43, 95%CI = 1.01–5.97), lacked development opportunities (adjOR = 5.97, 95%CI = 1.01–36.2), or were dissatisfied with career prospects (adjOR = 2.77, 95%CI = 1.04–7.27). Health system managers and relevant stakeholders involved in the planning, implementation, or evaluation of health policies and reforms aimed at improving healthcare job retention should take into account the key determinants of the intention to leave identified in this study.

## Introduction

Healthcare workforce shortages are a global issue. According to the World Health Organization (WHO), in 2022 there was a shortfall of approximately 1.2 million doctors, which is expected to worsen, with an estimated global shortage of nearly 14 million healthcare professionals, including nurses, physicians, midwives, and other essential roles, by 2030 [1].

The growing demand for physicians can be attributed to several factors, including an aging population, increasing prevalence of chronic diseases, and rising healthcare needs. These challenges have been further exacerbated by the COVID-19 pandemic, causing service delays [1]. Additionally, 40% of medical doctors are currently aged 55 or older, approaching retirement age, while workforce mobility and migration patterns complicate retention efforts [1]. As a result, many European countries are facing significant challenges in retaining physicians, within the healthcare sector. In light of this growing shortage of human resources, the ability to retain physicians within healthcare systems has become a strategic priority.

Retaining the medical workforce is essential for ensuring the sustainability and effectiveness of healthcare systems. Physician shortages, often exacerbated by resignations or decisions to leave the profession, have significant consequences. For patients, the lack of available physicians can result in longer wait times, reduced access to care, and compromised care quality [2]. For physicians currently at work, the departure of colleagues also increases their workload, contributing to stress, burnout, and further attrition [3]. For healthcare systems, the costs of training or recruiting new physicians are substantial [4]. High turnover also undermines efficiency and disrupts continuity of care.

Understanding the causes of turnover intention is crucial for is therefore essential to addressing the root causes of workforce shortages and effectively managing human resources in the healthcare sector and ensuring the quality and continuity of patient care [5]. Most studies on healthcare professionals’ turnover intention differentiate between leaving the hospital and leaving the profession. However, only a minority focus specifically on physicians. In a previous systematic review on turnover intentions by De Vries et al. [6], 78.4% of the studies focused exclusively on nurses, 10.8% examined both nurses and physicians, 2.7% involved healthcare professionals broadly, and only 8.1% focused solely on physicians.

Although the factors influencing the physicians’ intention to leave have been insufficiently studied, existing literature suggests that common reasons for doctors’ intention to change hospital include low job satisfaction, limited opportunities of career advancement, and excessive stress leading to burnout [6–8]. Other research highlights the impact of quantitative workload, which imposes high demands in terms of time, physical, emotional, and mental effort [9].

The COVID-19 pandemic has exacerbated the pressures on healthcare systems, contributing to increased stress, burnout, and incidents of violence against healthcare professionals, which has led many to leave their positions [10,11].

One study examined the intention to leave the hospital or profession for doctors in relation only to organizational context [12]. Others have analyzed how both burnout and job satisfaction are related to doctors’ intentions to leave their jobs [11,13]. Additional research considers well-being associated with work and elements pertaining to one’s career as some of the main factors in the intention to leave the profession [14].

While some studies have explored turnover intention in relation to organizational factors, job satisfaction, and burnout, few have considered both hospital and profession turnover simultaneously, especially when addressing the combined impact of work stressors [9]. Given these gaps in the literature, the present study aims to contribute new insights into the determinants of turnover intention among physicians of different European contexts.

This study aims to [1] estimate the prevalence of physicians expressing their intention to leave their hospital or the profession in Belgium, the Netherlands, Italy and Poland; and [2] analyze how job demands, job resources, job satisfaction, and burnout are associated to turnover intentions. The survey, conducted in 2022, offers a snapshot of physicians’ intention to leave their job in the period immediately following the COVID-19 pandemic.

## Materials and methods

### Survey methods

A cross-sectional multicentre study was conducted across four European Countries: Belgium, Netherlands, Italy, and Poland. Eight hospitals were selected, comprising one academic and one non-academic institution per country. Data were collected from May 16 to September 30, 2022. The sample size was calculated to estimate the proportion of physicians intending to leave their job, with a 95% confidence level and a 5% margin of error. Based on an estimated 28.2% turnover intention within two years [15], and accounting for a 20% non-response rate, the target sample size was 370 physicians. All physicians employed at each participating hospital as of April 1, 2022, were invited to participate. Invitations were sent via intranet or email by hospital contact persons, and contact details were kept confidential. A reminder email was sent after 14 days to encourage participation. If the response rate was lower than anticipated, medical associations were contacted to encourage further participation by explaining the potential benefits in terms of workplace well-being.

Before participating, respondents were required to read an information letter and provide online written informed consent. Inclusion criteria required participants to provide informed consent, receive adequate study information, and be currently employed as physicians at one of the eight participating hospitals. A complete description of the theoretical background and methods of the study is provided elsewhere [16]. The METEOR study protocol was approved by the Ethics Committee Research UZ/KU Leuven in January 2022 (S66009).

### Data collection

**Fig 1:**
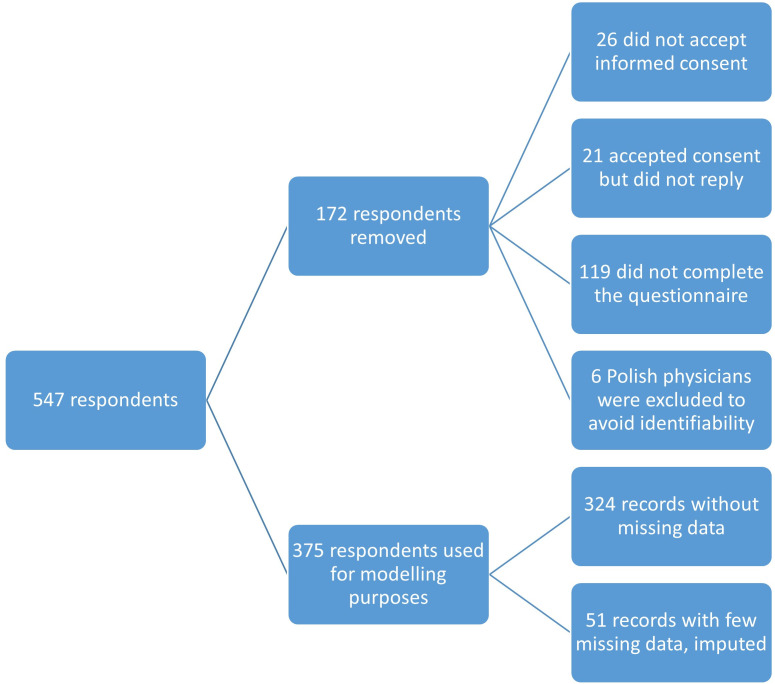
Flowchart of physicians’ data cleaning and imputation.

Of the 547 respondents to the invitation emails, 172 were excluded for the following reasons: 26 did not provide informed consent, 21 abandoned the questionnaire without answering any items, and 119 discontinued partway through. Furthermore, responses from Poland (n = 6) were excluded to ensure participant anonymity. This resulted in a final sample of 375 records, all of which were assessed for data quality, where no implausible response patterns were detected. These records were used for the descriptive and modeling analyses. Of the 375 cases, 324 were complete, while 51 contained missing data and were subsequently imputed (see Fig 1).

### Outcomes

The statements “I intend to leave my current hospital for another one in the near future” and “I intend to leave my healthcare profession for another job” were both scored on a 5-point Likert scale, ranging from “Strongly disagree” to “Strongly agree” [17]. The two outcomes were then dichotomized: a value of “1” was assigned if the answer was “Agree” or “Strongly agree” with the intention to leave the hospital (first outcome) or the healthcare profession (second outcome), and a value of “0” was assigned if they did not.

### Covariates

The covariates included in the analysis encompass a range of demographic characteristics (such as nationality, sex, age, and living status), work context factors (including workplace, form of employment, type of contract, nightshifts, lack of equipment, and ward of assignment), involvement as a frontline worker during the COVID-19 pandemic. Furthermore, burnout symptoms (divided into depersonalization and emotional exhaustion [18], job demands (covering staffing, work pace, quantitative, cognitive, and emotional demands, work-life conflicts, and role conflicts), bullying, job resources (such as influence, development, predictability, supervisor and colleagues’ support, meaning of work, and work engagement), and satisfaction (including prospects, physical working conditions, use of abilities, salary, and overall job satisfaction). Detailed information about the questionnaire can be found elsewhere [16,19]. The Emotional Exhaustion (EE) and Depersonalization (DP) items were rated on a 7-point Likert scale (0–6). The scores for each domain were summed to create composite scores: five DP items (0–6) yielded a total score ranging from 0 to 30, and nine EE items (0–6) yielded a total score ranging from 0 to 54. These composite scores were then dichotomized based on cut-offs, as suggested by Dall’Ora et al. [20]: a DP score ≥13 indicated “Yes” for Depersonalization, while a score <13 indicated “No.” Similarly, an EE score ≥27 indicated “Yes” for Emotional Exhaustion, while a score <27 indicated “No”. The remaining items were rated on a 5-point Likert scale and were dichotomized in the following way:

Agreement-based items: 1 for “Agree” and “Strongly agree”, and 0 for the other categories;

Frequency of occurrence: 1 for “Often” or “Always,” and 0 for the remaining categories;

Extent scale: 1 for “To a large extent” or “To a very large extent,” and 0 for the others;

Dissatisfaction scale: 1 for “Very unsatisfied” or “Unsatisfied,” and 0 for the remaining categories.

The variables in the job resources, work engagement, and job satisfaction sections were reverse-coded to reflect their lack or absence.

### Statistical analysis

Quantitative variables were summarized as means with standard deviations or as medians with interquartile ranges depending on the distribution of the data. Categorical variables were presented as counts and percentages. Missing data were imputed under the assumption of being “missing completely at random,” which was verified using Fisher’s exact test. Incomplete data records were imputed using the R package *mice*, which performs multiple imputation via chained equations [21]. Univariable and multivariable analyses were performed. The multivariable analysis included only variables that were statistically significant in the univariable analysis. A logistic regression model was fitted for each binary outcome of intention to leave, with covariates previous mentioned as independent variables. The analyses were adjusted for country, demographics and work context characteristics, including specialty (surgical vs. non-surgical), involvement in the recent epidemic (yes vs. no), and seniority. The multivariable logistic regression included only variables that were statistically significant in the univariable analysis. All analyses were conducted using R Statistical Software (v4.3.3; R Core Team, 2024), with p-values <0.05 considered statistically significant.

## Results

### The sample description

Of 375 physicians included in the final sample, most were from Belgium (42%), followed by the Netherlands (30%) and Italy (28%). In all countries, most respondents were female, except in Italy, where slightly more respondents were male (50.5%). The youngest physicians were from Belgium (mean age ± SD: 42 ± 10.5 years), while the oldest were from Italy (mean age ± SD: 47.3 ± 9.7 years). Overall, most physicians worked in non-surgical wards (78.9%) and lived with a partner and children (82.1%). More than half of the physicians were full-time (57.1%) and had permanent contracts (83.7%). The proportion of doctors assigned to night shifts varied, ranging from 29.7% in Belgian hospitals to 71.4% in the Netherlands. Over half of the physicians had served as frontline workers caring for COVID-19 patients (54.9%), with 66.5% of these frontline doctors engaged in this activity for more than one year. In general, most surveyed physicians (>80%) reported lacking all the equipment and consumable they needed to perform their jobs effectively. However, Italian respondents rated their equipment and consumable as adequate to a much lesser extent (39%). Eventually, 20.3% of physicians reported experiencing an illness, disability, or other physical or mental health problem caused or exacerbated by work (excluding occupational injuries) in the past three years. This ranged from 18.8% in the Netherlands to 21.5% in Belgium (Table 1).

**Table 1:** Individual characteristics of 375 physicians by country.

Characteristic	Overall	Belgium	Italy	Netherlands
	N = 375^1^	N = 158^1^	N = 105^1^	N = 112^1^
**Demographics**				
Gender				
*Female*	226 (60.3%)	96 (60.8%)	52 (49.5%)	78 (69.6%)
*Male*	149 (39.7%)	62 (39.2%)	53 (50.5%)	34 (30.4%)
Age (years)	44.8 (10.2)	42.0 (10.5)	47.3 (9.70)	46.3 (9.19)
Living status				
*Alone*	67 (17.9%)	25 (15.8%)	27 (25.7%)	15 (13.4%)
*With partner*	308 (82.1%)	133 (84.2%)	78 (74.3%)	97 (86.6%)
Health problems				
*No*	299 (79.7%)	124 (78.5%)	84 (80.0%)	91 (81.3%)
*Yes*	76 (20.3%)	34 (21.5%)	21 (20.0%)	21 (18.8%)
**Work context**				
Contract				
*Full-time*	214 (57.1%)	114 (72.2%)	100 (95.2%)	0 (0%)
*Part-time*	161 (42.9%)	44 (27.8%)	5 (4.8%)	112 (100%)
Employment				
*Permanent*	314 (83.7%)	135 (85.4%)	82 (78.1%)	97 (86.6%)
*Temporary*	61 (16.3%)	23 (14.6%)	23 (21.9%)	15 (13.4%)
Covid-19 frontline				
*No*	169 (45.1%)	76 (48.1%)	49 (46.7%)	44 (39.3%)
*Yes*	206 (54.9%)	82 (51.9%)	56 (53.3%)	68 (60.7%)
Nightshifts				
*No*	175 (46.7%)	111 (70.3%)	32 (30.5%)	32 (28.6%)
*Yes*	200 (53.3%)	47 (29.7%)	73 (69.5%)	80 (71.4%)
Lack of equipment				
*No*	74 (19.7%)	17 (10.8%)	41 (39.0%)	16 (14.3%)
*Yes*	301 (80.3%)	141 (89.2%)	64 (61.0%)	96 (85.7%)
Specialty				
*Non-surgery*	296 (78.9%)	121 (76.6%)	85 (81.0%)	90 (80.4%)
*Surgery*	79 (21.1%)	37 (23.4%)	20 (19.0%)	22 (19.6%)
Academic hospital				
*No*	138 (36.8%)	52 (32.9%)	39 (37.1%)	47 (42.0%)
*Yes*	237 (63.2%)	106 (67.1%)	66 (62.9%)	65 (58.0%)
**Burnout symptoms**				
Depersonalization				
*No*	354 (94.4%)	148 (93.7%)	99 (94.3%)	107 (95.5%)
*Yes*	21 (5.6%)	10 (6.3%)	6 (5.7%)	5 (4.5%)
Emotional exhaustion				
*No*	297 (79.2%)	122 (77.2%)	76 (72.4%)	99 (88.4%)
*Yes*	78 (20.8%)	36 (22.8%)	29 (27.6%)	13 (11.6%)
**Job demands**				
Poor staffing				
*No*	319 (85.1%)	137 (86.7%)	94 (89.5%)	88 (78.6%)
*Yes*	56 (14.9%)	21 (13.3%)	11 (10.5%)	24 (21.4%)
High working pace				
*No*	210 (56.0%)	103 (65.2%)	48 (45.7%)	59 (52.7%)
*Yes*	165 (44.0%)	55 (34.8%)	57 (54.3%)	53 (47.3%)
Quantitative demands				
*No*	251 (66.9%)	94 (59.5%)	89 (84.8%)	68 (60.7%)
*Yes*	124 (33.1%)	64 (40.5%)	16 (15.2%)	44 (39.3%)
Cognitive demands				
*No*	90 (24.0%)	40 (25.3%)	17 (16.2%)	33 (29.5%)
*Yes*	285 (76.0%)	118 (74.7%)	88 (83.8%)	79 (70.5%)
Emotional demands				
*No*	190 (50.7%)	87 (55.1%)	42 (40.0%)	61 (54.5%)
*Yes*	185 (49.3%)	71 (44.9%)	63 (60.0%)	51 (45.5%)
Work-life conflicts				
*No*	249 (66.4%)	97 (61.4%)	73 (69.5%)	79 (70.5%)
*Yes*	126 (33.6%)	61 (38.6%)	32 (30.5%)	33 (29.5%)
Role conflicts				
*No*	355 (94.7%)	146 (92.4%)	100 (95.2%)	109 (97.3%)
*Yes*	20 (5.3%)	12 (7.6%)	5 (4.8%)	3 (2.7%)
**Bullying**				
*No*	316 (84.3%)	126 (79.7%)	90 (85.7%)	100 (89.3%)
*Yes*	59 (15.7%)	32 (20.3%)	15 (14.3%)	12 (10.7%)
**Lack of job resources**				
Poor influence				
*No*	329 (87.7%)	133 (84.2%)	96 (91.4%)	100 (89.3%)
*Yes*	46 (12.3%)	25 (15.8%)	9 (8.6%)	12 (10.7%)
Poor development				
*No*	361 (96.3%)	156 (98.7%)	94 (89.5%)	111 (99.1%)
*Yes*	14 (3.7%)	2 (1.3%)	11 (10.5%)	1 (0.9%)
Poor predictability				
*No*	322 (85.9%)	138 (87.3%)	76 (72.4%)	108 (96.4%)
*Yes*	53 (14.1%)	20 (12.7%)	29 (27.6%)	4 (3.6%)
Poor supervisor support				
*No*	333 (88.8%)	133 (84.2%)	94 (89.5%)	106 (94.6%)
*Yes*	42 (11.2%)	25 (15.8%)	11 (10.5%)	6 (5.4%)
Poor colleagues support				
*No*	342 (91.2%)	141 (89.2%)	90 (85.7%)	111 (99.1%)
*Yes*	33 (8.8%)	17 (10.8%)	15 (14.3%)	1 (0.9%)
Poor meaning of work				
*No*	371 (98.9%)	157 (99.4%)	103 (98.1%)	111 (99.1%)
*Yes*	4 (1.1%)	1 (0.6%)	2 (1.9%)	1 (0.9%)
**Lack of work engagement**				
*No*	358 (95.5%)	152 (96.2%)	96 (91.4%)	110 (98.2%)
*Yes*	17 (4.5%)	6 (3.8%)	9 (8.6%)	2 (1.8%)
**Job dissatisfaction with**				
Work prospects				
*No*	302 (80.5%)	129 (81.6%)	83 (79.0%)	90 (80.4%)
*Yes*	73 (19.5%)	29 (18.4%)	22 (21.0%)	22 (19.6%)
Physical working conditions				
*No*	310 (82.7%)	142 (89.9%)	79 (75.2%)	89 (79.5%)
*Yes*	65 (17.3%)	16 (10.1%)	26 (24.8%)	23 (20.5%)
Use of abilities				
*No*	304 (81.1%)	133 (84.2%)	75 (71.4%)	96 (85.7%)
*Yes*	71 (18.9%)	25 (15.8%)	30 (28.6%)	16 (14.3%)
Salary				
*No*	274 (73.1%)	123 (77.8%)	54 (51.4%)	97 (86.6%)
*Yes*	101 (26.9%)	35 (22.2%)	51 (48.6%)	15 (13.4%)
Overall job dissatisfaction				
*No*	333 (88.8%)	140 (88.6%)	87 (82.9%)	106 (94.6%)
*Yes*	42 (11.2%)	18 (11.4%)	18 (17.1%)	6 (5.4%)

^1^ *n (%); Mean (SD).*

Regarding the estimated prevalence, the overall intention to leave (ITL) the hospital was 16.5% (95% CI: 13%–20%), with rates of 19.6% (95% CI: 13%–26%) in Belgium, 19% (95% CI: 11%–27%) in Italy, and 9.8% (95% CI: 4%–15%) in the Netherlands. The overall intention to leave the profession was 9.1% (95% CI: 6%–12%), with rates of 6.3% (95% CI: 3%–10%) in Belgium, 5.7% (95% CI: 1%–10%) in Italy, and 16.1% (95% CI: 9%–23%) in the Netherlands (S1 Table).

### Univariable analysis

Regarding the intention to leave the hospital, several factors were identified as significant predictors. Age was negatively associated with the likelihood of leaving (OR: 0.92, 95% CI: 0.89–0.95, p < 0.001), as well as living with a partner (vs. living alone) (OR: 0.50, 95% CI: 0.27–0.97, p = 0.04) and having a part-time job (OR: 0.37, 95% CI: 0.19–0.67, p < 0.001). Conversely, having a temporary job increased the probability of leaving (OR: 2.07, 95% CI: 1.06–3.92, p = 0.03). Physicians in the Netherlands were less likelihood of leaving the hospital compared to Belgian physicians (OR: 0.45, 95% CI: 0.21–0.91, p = 0.03). Both depersonalization (OR: 3.42, 95% CI: 1.30–8.51, p = 0.01) and emotional exhaustion (OR: 3.62, 95% CI: 2.01–6.51, p < 0.001) were strong risk factors for the intention to leave. Among the components of job demands, work-life conflict (OR: 1.96, 95% CI: 1.12–3.40, p = 0.01) and role conflict (OR: 3.72, 95% CI: 1.40–9.42, p = 0.01) significantly increased the likelihood of leaving. For job resources, negative factors such as poor influence at work (OR: 3.28, 95% CI: 1.63–6.43, p = 0.01), lack of development opportunities (OR: 4.08, 95% CI: 1.30–12.2, p = 0.01), lack of predictability (OR: 4.04, 95% CI: 2.10–7.66, p < 0.001), poor supervisor support (OR: 2.60, 95% CI: 1.23–5.26, p = 0.01), poor colleague support (OR: 3.34, 95% CI: 1.51–7.12, p = 0.01), lack of meaning in work (OR: 6.47, 95% CI: 2.37–18.0, p < 0.001), and lack of work engagement (OR: 15.9, 95% CI: 1.99–3.24, p = 0.01) were highly associated with the intention to leave. Finally, dissatisfaction with work-related aspects also emerged as strong predictors of intention to leave. These included dissatisfaction with work prospects (OR: 4.48, 95% CI: 2.47–8.10, p < 0.001), dissatisfaction with the use of one’s abilities (OR: 3.92, 95% CI: 2.15–7.11, p < 0.001), dissatisfaction with salary (OR: 2.11, 95% CI: 1.18–3.71, p = 0.01), and overall dissatisfaction (OR: 5.57, 95% CI: 2.79–11.1, p < 0.001).

Regarding the intention to leave the profession, physicians in the Netherlands had a significantly higher likelihood of agreeing to change their profession compared to those in Belgium (OR: 2.83, 95% CI: 1.28–6.63, p = 0.01). Several factors were associated with an increased risk of leaving the profession, including working in a surgical ward (OR: 3.42, 95% CI: 1.63–7.08, p = 0.01), performing nightshifts (OR: 3.12, 95% CI: 1.43–7.55, p = 0.01), and having experienced health problems in the past (OR: 3.15, 95% CI: 1.48–6.54, p = 0.01). In contrast, working in an academic hospital appeared to be a protective factor (OR: 0.48, 95% CI: 0.24–0.98, p = 0.04). Experiencing depersonalization (OR: 4.66, 95% CI: 1.56–12.5, p = 0.01) and emotional exhaustion (OR: 2.63, 95% CI: 1.22–5.47, p = 0.01) were strongly associated with an increased probability of leaving the profession. Among job demands, only quantitative demands (OR: 2.85, 95% CI: 1.40–5.90, p = 0.01), work-life conflict (OR: 2.13, 95% CI: 1.04–4.35, p = 0.04), and role conflict (OR: 3.75, 95% CI: 1.15–10.5, p = 0.03) significantly increased the likelihood of leaving the profession. Finally, job resource deficits were notable risk factors, including the lack of development opportunities (OR: 6.36, 95% CI: 1.85–19.7, p = 0.01) and lack of predictability (OR: 2.89, 95% CI: 1.24–6.31, p = 0.01) (Table 2).

**Table 2:** Univariable analysis for physicians’ intention to leave: Results from logistic regression.

Characteristics^1^	Intention to leave the hospital					Intention to leave the profession				
	No	Yes	OR	95%CI	P	No	Yes	OR	95%CI	P
**Demographics**										
Gender										
*Female*	195 (86%)	31 (14%)	–	–	–	209 (92.5%)	17 (7.5%)	–	–	–
*Male*	118 (79%)	31 (21%)	1.65	0.95, 2.86	0.07	132 (89%)	17 (11%)	1.58	0.78, 3.23	0.20
Age										
*Mean (SD)*	45 (40.5)	36 (29.5)	0.92	0.89, 0.95	**<0.001**	44 (38.5)	45 (37.5)	0.98	0.95, 1.02	0.40
Country, n (%)										
*Belgium*	127 (80%)	31 (20%)	–	–	–	148 (94%)	10 (6.3%)	–	–	–
*Italy*	85 (81%)	20 (19%)	0.96	0.51, 1.79	0.90	99 (94%)	6 (5.7%)	0.90	0.30, 2.49	0.83
*Netherlands*	101 (90%)	11 (9.8%)	0.45	0.21, 0.91	**0.03**	94 (84%)	18 (16%)	2.83	1.28, 6.63	**0.01**
Living status										
*Alone*	50 (75%)	17 (25%)	–	–	–	59 (88%)	8 (12%)	–	–	–
*With partner and children*	263 (85%)	45 (15%)	0.50	0.27, 0.97	**0.04**	282 (92%)	26 (8.4%)	0.68	0.30, 1.67	0.38
Health problems										
*No*	253 (85%)	46 (15%)	–	–	–	279 (93%)	20 (7%)	–	–	–
*Yes*	60 (79%)	16 (21%)	1.47	0.76, 2.72	0.25	62 (82%)	14 (18%)	3.15	1.48, 6.54	**0.01**
**Work context**										
Contract										
*Full-time*	167 (78%)	47 (22%)	–	–	–	200 (93%)	14 (6.5%)	–	–	–
*Part-time*	146 (91%)	15 (9.3%)	0.37	0.19, 0.67	**<0.001**	141 (88%)	20 (12%)	2.03	1.00, 4.23	0.05
Employment										
*Permanent*	268 (85%)	46 (15%)	–	–	–	285 (91%)	29 (9.2%)	–	–	–
*Temporary*	45 (74%)	16 (26%)	2.07	1.06, 3.92	**0.03**	56 (92%)	5 (8.2%)	0.88	0.29, 2.19	0.79
Covid frontline										
*No*	141 (83%)	28 (17%)	–	–	–	157 (93%)	12 (7%)	–	–	–
*Yes*	172 (83%)	34 (17%)	1.00	0.58, 1.73	0.99	184 (89%)	22 (11%)	1.56	0.76, 3.36	0.23
Nightshifts										
*No*	141 (81%)	34 (19%)	–	–	–	167 (95%)	8 (5%)	–	–	–
*Yes*	172 (86%)	28 (14%)	0.68	0.39, 1.17	0.16	174 (87%)	26 (13%)	3.12	1.43, 7.55	**0.01**
Lack of equipment										
*No*	57 (77%)	17 (23%)	–	–	–	63 (85%)	11 (15%)	–	–	–
*Yes*	256 (85%)	45 (15%)	0.59	0.32, 1.13	0.11	278 (92%)	23 (7.6%)	0.47	0.22, 1.06	0.06
Specialty										
*Non-surgery*	253 (85%)	43 (15%)	–	–	–	277 (94%)	19 (6.4%)	–	–	–
*Surgery*	60 (76%)	19 (24%)	1.86	1.00, 3.39	0.05	64 (81%)	15 (19%)	3.42	1.63, 7.08	**0.01**
Academic hospital										
*No*	115 (83%)	23 (17%)	–	–	–	120 (87%)	18 (13%)	–	–	–
*Yes*	198 (84%)	39 (16%)	0.98	0.56, 1.75	0.96	221 (93%)	16 (6.8%)	0.48	0.24, 0.98	**0.04**
**Burnout**										
Depersonalization										
*No*	300 (85%)	54 (15%)	3.42	1.30, 8.51	**0.01**	326 (92%)	28 (7.9%)	4.66	1.56, 12.5	**0.01**
*Yes*	13 (62%)	8 (38%)				15 (71%)	6 (29%)			
Emotional exhaustion										
*No*	261 (88%)	36 (12%)	–	–	–	276 (93%)	21 (7.1%)	–	–	–
*Yes*	52 (67%)	26 (33%)	3.62	2.01, 6.51	**<0.001**	65 (83%)	13 (17%)	2.63	1.22, 5.47	**0.01**
**Job demands**										
Poor staffing										
*No*	263 (82%)	56 (18%)	–	–	–	290 (91%)	29 (9.1%)	–	–	–
*Yes*	50 (89%)	6 (11%)	0.56	0.21, 1.29	0.18	51 (91%)	5 (8.9%)	0.98	0.32, 2.45	0.97
High working pace										
*No*	169 (80%)	41 (20%)	–	–	–	187 (89%)	23 (11%)	–	–	–
*Yes*	144 (87%)	21 (13%)	0.60	0.33, 1.05	0.07	154 (93%)	11 (6.7%)	0.58	0.26, 1.20	0.15
Quantitative demands										
*No*	209 (83%)	42 (17%)	–	–	–	236 (94%)	15 (6.0%)	–	–	–
*Yes*	104 (84%)	20 (16%)	0.96	0.53, 1.69	0.08	105 (85%)	19 (15%)	2.85	1.40, 5.90	**0.01**
Cognitive demands										
*No*	71 (79%)	19 (21%)	–	–	–	84 (93%)	6 (6.7%)	–	–	–
*Yes*	242 (85%)	43 (15%)	0.66	0.37, 1.23	0.19	257 (90%)	28 (9.8%)	1.53	0.65, 4.19	0.35
Emotional demands										
*No*	161 (85%)	29 (15%)	–	–	–	173 (91%)	17 (8.9%)	–	–	–
*Yes*	152 (82%)	33 (18%)	1.21	0.70, 2.09	0.50	168 (91%)	17 (9.2%)	1.03	0.51, 2.10	0.94
Work-life conflicts										
*No*	216 (87%)	33 (13%)	–	–	–	232 (93%)	17 (6.8%)	–	–	–
*Yes*	97 (77%)	29 (23%)	1.96	1.12, 3.40	**0.01**	109 (87%)	17 (13%)	2.13	1.04, 4.35	**0.03**
Role conflicts										
*No*	301 (85%)	54 (15%)	–	–	–	326 (92%)	29 (8.2%)	–	–	–
*Yes*	12 (60%)	8 (40%)	3.72	1.40, 9.42	**0.01**	15 (75%)	5 (25%)	3.75	1.15, 10.5	**0.04**
**Bullying**										
*No*	264 (84%)	52 (16%)	–	–	–	289 (91%)	27 (8.5%)	–	–	–
*Yes*	49 (83%)	10 (17%)	1.04	0.47, 2.10	0.93	52 (88%)	7 (12%)	1.44	0.55, 3.32	0.43
**Lack of job resources**										
Poor influence										
*No*	283 (86%)	46 (14%)	–	–	–	303 (92%)	26 (7.9%)	–	–	–
*Yes*	30 (65%)	16 (35%)	3.28	1.63, 6.43	**0.01**	38 (83%)	8 (17%)	2.45	0.98, 5.60	0.05
Poor development										
*No*	305 (84%)	56 (16%)	–	–	–	332 (92%)	29 (8.0%)	–	–	–
*Yes*	8 (57%)	6 (43%)	4.08	1.30, 12.2	**0.01**	9 (64%)	5 (36%)	6.36	1.85, 19.7	**0.01**
Poor predictability										
*No*	280 (87%)	42 (13%)	–	–	–	298 (93%)	24 (7.5%)	–	–	–
*Yes*	33 (62%)	20 (38%)	4.04	2.10, 7.66	**<0.001**	43 (81%)	10 (19%)	2.89	1.24, 6.31	**0.01**
Poor supervisor support										
*No*	284 (85%)	49 (15%)	–	–	–	306 (92%)	27 (8.1%)	–	–	–
*Yes*	29 (69%)	13 (31%)	2.60	1.23, 5.26	**0.01**	35 (83%)	7 (17%)	2.27	0.86, 5.35	0.09
Poor colleagues support										
*No*	292 (85%)	50 (15%)	–	–	–	313 (92%)	29 (8.5%)	–	–	–
*Yes*	21 (64%)	12 (36%)	3.34	1.51, 7.12	**0.01**	28 (85%)	5 (15%)	1.93	0.62, 5.01	0.24
Poor meaning of work										
*No*	312 (84%)	59 (16%)	–	–	–	338 (91%)	33 (8.9%)	–	–	–
*Yes*	1 (25%)	3 (75%)	6.47	2.37, 18.0	**<0.001**	3 (75%)	1 (25%)	3.41	0.17, 27.5	0.35
**Lack of work engagement**										
*No*	305 (85%)	53 (15%)	–	–	–	330 (92%)	28 (7.8%)	–	–	–
*Yes*	8 (47%)	9 (53%)	15.9	1.99, 3.24	**0.01**	11 (65%)	6 (35%)	6.43	2.08, 18.3	**0.01**
**Job dissatisfaction with**										
Work prospects										
*No*	267 (88%)	35 (12%)	–	–	–	286 (95%)	16 (5.3%)	–	–	–
*Yes*	46 (63%)	27 (37%)	4.48	2.47, 8.10	**<0.001**	55 (75%)	18 (25%)	5.85	2.81, 12.3	**<0.001**
Physical working conditions										
*No*	263 (85%)	47 (15%)	–	–	–	291 (94%)	19 (6.1%)	–	–	–
*Yes*	50 (77%)	15 (23%)	1.68	0.85, 3.18	0.13	50 (77%)	15 (23%)	4.59	2.17, 9.63	**<0.001**
Use of abilities										
*No*	267 (88%)	37 (12%)	–	–	–	282 (93%)	22 (7.2%)	–	–	–
*Yes*	46 (65%)	25 (35%)	3.92	2.15, 7.11	**<0.001**	59 (83%)	12 (17%)	2.61	1.19, 5.48	**0.01**
Salary										
*No*	237 (86%)	37 (14%)	–	–	–	250 (91%)	24 (8.8%)	–	–	–
*Yes*	76 (75%)	25 (25%)	2.11	1.18, 3.71	**0.01**	91 (90%)	10 (9.9%)	1.14	0.51, 2.42	0.73
Overall job dissatisfaction										
*No*	290 (87%)	43 (13%)	–	–	–	310 (93%)	23 (6.9%)	–	–	–
*Yes*	23 (55%)	19 (45%)	5.57	2.79, 11.1	**<0.001**	31 (74%)	11 (26%)	4.78	2.07, 10.6	**<0.001**

^1^For each variable, the first category is the reference one; Significant p-values are highlighted in bold-type. *The OR for Age is meant with respect to one-year increases.

### Multivariable analysis

Estimates from multivariable models for both the intention to leave the hospital and the profession are presented in Table 3.

**Table 3:** Multivariable analysis for physicians’ intention to leave: Results from logistic regression.

Characteristics	Intention to leave the hospital			Intention to leave the profession		
	aOR	95%CI	P	aOR	95%CI	P
**Demographics**						
Sex (Male vs Female)	1.84	0.95, 3.58	0.07	1.99	0.79, 5.04	0.14
Age	0.90	0.86, 0.93	**<0.001**	0.96	0.91, 1.00	0.07
Country (vs Belgium)						
*Italy*	–	–	–	0.35	0.07, 1.37	0.20
*Netherlands*	–	–	–	4.14	1.62, 11.4	**0.01**
Living status (With partner vs Alone)	0.56	0.27, 1.20	0.13	–	–	–
**Work context**						
Contract (Part-time vs Full-time)	0.54	0.25, 1.10	0.10	–	–	–
Specialty (Surgery)	–	–	–	2.90	1.22, 6.78	**0.01**
Academic hospital	–	–	–	0.41	0.17, 0.99	**0.04**
**Job demands**						
Quantitative demands	–	–	–	2.16	0.90, 5.26	0.08
**Lack of job resources**						
Poor development	–	–	–	5.97	1.01, 36.2	**0.04**
Poor colleagues support	3.18	1.06, 9.36	**0.03**	–	–	–
**Job dissatisfaction with**						
Work prospects	2.38	1.02, 5.54	**0.04**	2.77	1.04, 7.27	**0.03**
Physical working conditions	–	–	–	2.62	0.95, 7.08	0.05
Use of abilities	1.86	0.83, 4.07	0.12	–	–	–
Overall job dissatisfaction	2.71	1.09, 6.69	**0.03**	2.73	0.86, 8.45	0.08

The first model highlights that younger physicians (adjOR = 0.90, 95% CI: 0.86–0.93, p < 0.001), those with poor support from colleagues (adjOR = 3.18, 95% CI: 1.06–9.36, p = 0.03), those dissatisfied with work prospects (adjOR = 2.38, 95% CI: 1.02–5.54, p = 0.04), and those reporting overall job dissatisfaction (adjOR = 2.71, 95% CI: 1.09–6.69, p = 0.03) are more likely to intend to leave the hospital.

The second model indicates that physicians from the Netherlands (adjOR = 4.14, 95% CI: 1.62–11.4, p = 0.01), surgeons (adjOR = 2.90, 95% CI: 1.22–6.78, p = 0.01), those working in non-academic hospitals (adjOR = 0.41, 95% CI: 0.17–0.99, p = 0.04), those lacking development opportunities (adjOR = 5.97, 95% CI: 1.01–36.2, p = 0.04), and those dissatisfied with work prospects (adjOR = 2.77, 95% CI: 1.04–7.27, p = 0.03) are more likely to intend to leave the profession.

## Discussion

This study addressed gaps in research by exploring factors influencing physicians’ intention to leave the hospital and the medical profession. The results were analyzed focusing on individual, social, and organizational aspects. In the first part of the research, factors related to the intention to leave the hospital were explored, while the second part focused on the intention to leave the profession. The findings highlight significant differences both at the individual level and in relation to the cultural and organizational context.

### Intention to leave the hospital

The key factors associated with the intention to leave the hospital include age, support from colleagues, career prospects, and overall job satisfaction. The results suggest that younger physicians are more likely to consider leaving the hospital, which is in line with previous studies indicating that limited work experience may reduce resilience to stress [6]. Young physicians, early in their careers, show a greater openness to career change, driven by high expectations, personal ambitions, and the search for opportunities that can enhance their skills and accelerate professional growth [22,23]. Their young age also makes them more sensitive to unfavorable working conditions, such as excessive hours, work-life imbalance, and lack of organizational support, leading them to leave positions that do not meet their standards more easily [24]. Their greater flexibility and ambition drive them to consider opportunities in more prestigious settings or abroad, facilitated by a lower risk aversion due to the absence of binding family or financial ties [25].

Secondly, insufficient support from colleagues has been identified as a significant determinant, contributing to high levels of burnout and dissatisfaction. A collaborative environment and support from colleagues and superiors significantly reduce stress and burnout, as demonstrated by West et al. (2018), who emphasize that empathetic leadership and an organizational culture based on mutual support improve job satisfaction, thereby reducing turnover intentions [26].

Lastly, the lack of professional prospects and adequate career progression has also been correlated with a higher intention to leave the hospital, suggesting that the perception of professional stagnation fuels dissatisfaction and the decision to seek new job opportunities. The possibility of professional advancement is crucial for maintaining physicians’ motivation [27]. Thus, overall job dissatisfaction emerges as one of the strongest indicators of the intention to leave the hospital, reflecting difficulties related to excessive workload, a stressful environment, and scarce resources [27].

### Intention to leave the profession

Regarding the intention to leave the profession, the results show that differences between countries play a fundamental role. The difference in the intention to leave rates between Belgium, the Netherlands, and Italy could be attributed to cultural, social, and political factors. Specifically, in Italy, the labor market is characterized by stricter regulations, furthermore doctors face a chronic staff shortage and hospital service overload, compounded by inadequate healthcare policies and an aging workforce, which, combined with a highly bureaucratic system, limits career opportunities and fuels dissatisfaction [28]. In the Netherlands, despite having an efficient healthcare system, burnout among doctors is high due to high work expectations and the pressure to balance care quality with economic sustainability [29]. Belgian physicians also experience significant workloads. However, the country’s organized healthcare system, the emphasis on work-life balance and the opportunities for meaningful professional engagement appear to mitigate higher rates of professional abandonment [30].

In our study, surgeons were found at a higher risk of leaving the profession compared to other specialists, probably due to the intense workloads, high psychological and physical demands, and limited professional opportunities [31]. One study highlights that surgeons experience high levels of burnout, with a significant percentage considering leaving due to stress. Factors such as long working hours, high decision-making pressure, and huge responsibilities contribute to this phenomenon [32]. Additionally, a systematic review found that burnout increases the risk of medical or surgical errors by 2.5 times, contributing to a higher likelihood of surgeons considering leaving the profession [33].

Moreover, working in an academic hospital has proven to be a protective factor. University doctors are less likely to leave the profession due to the combination of clinical, teaching, and research activities, which stimulate professional growth. Additionally, the academic environment offers greater work flexibility and a more stimulating context, confirmed by a study where doctors involved in teaching and research report lower levels of burnout [34].

However, dissatisfaction related to the lack of professional development and limited career prospects were significant determinants of the intention to leave the profession. This aligns with the literature suggesting that the absence of advancement opportunities is one of the main causes of burnout and turnover [35]. Career development and progression opportunities are key, and a lack of these is a common reason for leaving the workforce in most countries [36].

Finally, the COVID-19 pandemic was an extraordinary event that amplified burnout and frustration among physicians, contributing to an increase in the intention to leave the profession or the hospital setting. As highlighted in a systematic review, the pandemic crisis exacerbated issues already entrenched in global healthcare systems, leading to unprecedented levels of stress and dissatisfaction among medical and nursing staff [36]. Although COVID-19 was not found to be significant in the current study, it is worth noting that the same study was conducted during the pandemic.

### Strengths and limitations

The first strength lies in the composition of the study sample. Although the sample was not randomly selected, the countries included were intentionally chosen to reflect the diversity within the European Union, capturing variations in geography, healthcare systems, and GDP per capita, thereby offering a snapshot of the EU’s intrinsic heterogeneity. Furthermore, another major strength of our study was its focus on the most relevant factors associated with the intention to leave the hospital and the profession.

A key limitation of this study is the low response rate, which may introduce response bias and limit the generalizability of the findings. Respondents may differ systematically from non-respondents—for example, being more engaged or motivated—which could lead to an overrepresentation of certain perspectives, while individuals with less favorable experiences might have been underrepresented. Furthermore, the exclusion of Polish data—given the country’s size and potential differences—reduces the representativeness of the European context and may bias cross-country comparisons. As such, observed differences between countries should be interpreted with caution, as they may reflect variations in response patterns as much as true underlying differences.

Then, the cross-sectional approach does not allow for monitoring changes over time nor establishing causal relationships between factors like burnout and the intention to leave. Future studies could adopt a longitudinal approach to track how the intention to leave evolves over time and how changes in working conditions, healthcare policies, or support interventions influence this dynamic. Furthermore, future studies could include general practitioners emphasizing the specific challenges faced by these groups.

## Implication for future research

Further investigation is needed into the effectiveness of targeted retention strategies, such as mentorship programs, improving organizational support, work flexibility, and investments in continuous education, including general practitioners and other extra-hospital professionals for a more comprehensive understanding. Additionally, deeper investigation of how cultural, social, and political factors influence abandonment rates across countries could provide valuable insights for the development of targeted healthcare policies at the national level.

## Conclusion

This study highlights the importance of both individual, social and organizational variables in determining the intention to leave the hospital and the medical profession. Factors such as age, support from colleagues, and career prospects are central to healthcare workers’ retention, while national variables and the type of hospital (academic or not) influence turnover intentions. The findings suggest that retention policies should focus on improving social support within hospitals, expanding career opportunities and professional development, as well as creating work environments that promote psychological and physical well-being. Investing in the retention of medical personnel, through targeted interventions to improve well-being at work, provide psychological support and promote a better work-life balance, is essential to ensure a resilient healthcare system responsive to population needs.

## Supporting information

S1 Table This is the S1 Table Overall, and by Country ITL of survey’s respondents, with 95% CI for the prevalence of ITL. (DOCX)

S2 Table This is the S2 Table Goodness-of-fit indices for the logistic regression models. (DOCX)
