# Supplementary material for: Stay or go? Exploring physician turnover in European Hospitals–Evidence from the METEOR survey
Source: PLoS One. 2025 Nov 21;20(11):e0337287. doi: 10.1371/journal.pone.0337287 (PMC12637990; doi:10.1371/journal.pone.0337287)
Supplement: S1 Table — (DOCX) [file pone.0337287.s001.docx]

| *Table S1. Overall, and by Country ITL of survey’s respondents, with 95% CI for the prevalence of ITL.* | | | | | | | | | | |
| --- | --- | --- | --- | --- | --- | --- | --- | --- | --- | --- |
|  |  | Intention to leave the hospital | | | Intention to leave the profession | | |  |  |  |
| **Country** | **N** | Yes | % | 95% CI | Yes | % | 95% CI |  |  |  |
| Belgium | 158 | 31 | 19.6 | 13.7-26.7 | 10 | 6.3 | 3.0-11.3 |  |  |  |
| Italy | 105 | 20 | 19 | 12.0-27.9 | 6 | 5.7 | 2.0-12.0 |  |  |  |
| Netherlands | 112 | 11 | 9.8 | 5.0-16.9 | 18 | 16.1 | 9.8-24.0 |  |  |  |
| Overall | 375 | 62 | 16.5 | 12.9-20.1 | 34 | 9.1 | 6.4-12.4 |  |  |  |
