# Supplementary material for: Stay or go? Exploring physician turnover in European Hospitals–Evidence from the METEOR survey
Source: PLoS One. 2025 Nov 21;20(11):e0337287. doi: 10.1371/journal.pone.0337287 (PMC12637990; doi:10.1371/journal.pone.0337287)
Supplement: S2 Table — (DOCX) [file pone.0337287.s002.docx]

*Table S2. Goodness-of-fit indices for the logistic regression models*

| **Model** | | **AIC** | **BIC** | | **Pseudo R² (Nagelkerke)** |
| --- | --- | --- | --- | --- | --- |
| Model 1 | Intention to leave the hospital | 268.95 | 300.36 | 0.33 | |
| Model 2 | Intention to leave the profession | 193.64 | 228.97 | 0.28 | |
